# Engaging stakeholders in research to address water–energy–food (WEF) nexus challenges

**DOI:** 10.1007/s11625-018-0552-7

**Published:** 2018-04-04

**Authors:** C. Hoolohan, A. Larkin, C. McLachlan, R. Falconer, I. Soutar, J. Suckling, L. Varga, I. Haltas, A. Druckman, D. Lumbroso, M. Scott, D. Gilmour, R. Ledbetter, S. McGrane, C. Mitchell, D. Yu

**Affiliations:** 1 Tyndall Centre for Climate Change Research, University of Manchester, Manchester, UK; 2 University of Abertay Dundee, Dundee, UK; 3 University of Exeter, Exeter, UK; 4 University of Surrey, Guildford, UK; 5 Cranfield University, Cranfield, UK; 6 HR Wallingford, Wallingford, UK; 7 University of Glasgow, Glasgow, UK; 8 Loughborough University, Loughborough, UK

**Keywords:** Water, Energy, Nexus, Transdisciplinary, Mixed-method

## Abstract

The water–energy–food (WEF) nexus has become a popular, and potentially powerful, frame through which to analyse interactions and interdependencies between these three systems. Though the case for transdisciplinary research in this space has been made, the extent of stakeholder engagement in research remains limited with stakeholders most commonly incorporated in research as end-users. Yet, stakeholders interact with nexus issues in a variety of ways, consequently there is much that collaboration might offer to develop nexus research and enhance its application. This paper outlines four aspects of nexus research and considers the value and potential challenges for transdisciplinary research in each. We focus on assessing and visualising nexus systems; understanding governance and capacity building; the importance of scale; and the implications of future change. The paper then proceeds to describe a novel mixed-method study that deeply integrates stakeholder knowledge with insights from multiple disciplines. We argue that mixed-method research designs—in this case orientated around a number of cases studies—are best suited to understanding and addressing real-world nexus challenges, with their inevitable complex, non-linear system characteristics. Moreover, integrating multiple forms of knowledge in the manner described in this paper enables research to assess the potential for, and processes of, scaling-up innovations in the nexus space, to contribute insights to policy and decision making.

## Introduction

The water–energy–food (WEF) nexus has become a popular framing for research that aims to explore the interactions and interdependencies between these three resource domains. It is frequently used in the context of research that spans disciplines, and has been argued to offer a new route into research that helps to address global societal challenges such as food security and climate change. Yet, questions have been raised as to whether this packaging offers anything new to the literature (Benson et al. 2015; Leck et al. 2015), and perhaps more importantly, whether it assists with bridging the gap between academic research, and the challenges faced by non-academic organisations. Indeed, it is arguably the case that non-academic organisations have been working across the water–energy–food nexus in practice, and for some time, without needing to name it as such. So, there is potentially much to be learnt through interaction with stakeholders operating in this space.

Recognising the importance of stakeholder’s experiences has led to transdisciplinary research gaining popularity in nexus research, at least in theory (Stirling 2015). As well as incorporating multiple academic disciplines, transdisciplinary research aspires to explicitly include non-academic stakeholders as co-creators of knowledge (Harris and Lyon 2013). Transdisciplinary projects are beginning to emerge in the nexus space, in some cases deliberately shaped and incentivised by research councils such as the projects funded through the UK’s Economic and Social Research Council’s (ESRC) Nexus Network and the Engineering and Physical Science Research Council’s (EPSRC) Water Energy Food Nexus Sandpit. Each of these streams sought to fund projects that positioned non-academic stakeholders as collaborators in research, whose understanding of nexus-related challenges could improve the integrity and validity of research, and through whom research might have material impact as well as academic relevance.

In this paper, we reflect on recent progress in stakeholder engagement in nexus-related research. We outline four emerging themes within nexus research and consider the value of, and potential challenges for, transdisciplinary research in each. Specifically, we examine how transdisciplinary approaches are used in assessing and visualising nexus issues; understanding governance and building capacity; accounting for multi and inter-scalar relationships; and exploring the implications of future social, technological and climatic change. Existing research in these four areas is discussed with regards to the involvement and inclusion of stakeholder knowledge in Sect. 2. We argue that stakeholder engagement within the nexus research agenda is as yet limited despite widespread recognition of the potential value of transdisciplinary research, with non-academic partners typically positioned as end-users of academic research rather than co-creators of knowledge. Subsequently, there remains scope to develop transdisciplinary methods within the context of nexus research.

To contribute towards research development in this area, we outline the research design developed for the Stepping Up project in Sect. 3. Stepping Up was one of three projects funded through the EPSRC sandpit, that from the outset sought to embed stakeholder knowledges throughout the research (RCUK 2015), to sufficiently ground research questions and findings in their practical experiences. Here we describe Stepping Up’s unique mixed method research design, through which an ongoing dialogue with stakeholders from a range of fields was developed, allowing the research team to connect qualitative insights with quantitative model outputs. We propose that a mixed method research design, such as that described, is the most effective means of accommodating the complex, non-linearity of nexus challenges and provides multiple opportunities for the inclusion of stakeholders as co-creators of knowledge to increase the efficacy of the research and enable better representation of real-world processes of innovations in the WEF nexus space.

## Research challenges in the WEF nexus space

### Assessing and visualising nexus issues

The principal aim of much of the nexus research is to develop tools to assess and communicate the connections and interdependencies between the three component systems. There are many ways to classify existing tools and for the purpose of this discussion, we examine those that cover at least two of the three WEF sectors and are widely accessible or open access (see Ferroukhi et al. 2015 for review), classifying them in terms of their objectives: sustainability assessment, modelling (including optimisation), and visualisation (Endo et al. 2015). There is broad recognition that involving stakeholders in the development of such tools may enhance their effectiveness, and a number of studies that specifically demonstrate how transdisciplinary methods facilitate a shared understanding that aids the design of appropriate interventions (Verweij and Thompson 2006; Blackwood et al. 2014) and encourage trust in decision support tools (Howarth and Monasterolo 2017). However, the inclusion of stakeholders in the development of nexus tools has as yet been limited.

Sustainability assessments, such as those that provide descriptions of resource use and availability across the WEF sectors, have been a major focus of nexus research to-date (e.g., Bajželj et al. 2014; Hatfield-Dodds et al. 2015). Many of the resulting models are quantitative in nature, and engage with stakeholders only as end-users of the technical information they provide. However, Flammini (2014) demonstrated the value of a mixed-method approach that embedded stakeholder input throughout the assessment process. Using qualitative and quantitative methods, this approach combined stakeholder input with national datasets and country typologies (e.g., dry country with agricultural-based economy) to produce an accessible framework for the rapid appraisal of the baseline and existing pressures faced in a given location. The dedicated inclusion of stakeholders in this process is shown to extend the scope of the assessment, incorporating the expectations, objectives and understandings of different parties to better represent nexus interlinkages. Similar approaches are emerging across the nexus space using mixed (e.g., Endo et al. 2015), and qualitative (e.g., Byrne 2016) methods alongside quantitative; and seeking stakeholder input in the development of decision support tools. Developing a two way science-policy interface in this way has been shown to better accommodate the complexity of the nexus in sustainability assessment, and enhance the application of research (Howarth and Monasterolo 2017).

Optimisation tools are used to explore policy and management options, structuring nexus problems as the culmination of multiple, often conflicting, criteria with the principal objective being to identify the best solution from a set of alternative management options. Numerous optimisation methods have been used in nexus research; approaches based on sustainability indicators are common, where weighting and ranking of indicators are sought from decision makers and combined using Multi Criteria Decision Analysis (MCDA). MCDA is known to be subjective, requiring value judgements that become implicit in model outputs (Flammini 2014) and, as an alternative, Multi-Objective Optimisation (MOO) addresses this subjectivity by expressing decision makers’ preferences following optimisation. Considerable progress has been made in applying MOO in nexus research (Lautenbach et al. 2012; Hurford et al. 2014) and research into its constituent systems (e.g., Reed et al. 2013).

The technical complexity and computational requirements of MOO reduce the accessibility of these tools to non-academic parties. Further optimisation does not always provide practical solutions, as uncertainties surrounding future conditions can leave optimal solutions vulnerable to failure, and practitioners may seek near-optimal solutions that address objectives beyond the scope of the model (Rosenberg 2015). Uncertainty can be partially addressed by coupling MOO with deep uncertainty approaches to assess the performance of potential interventions under different plausible futures (Herman et al. 2014). Such approaches are as yet underdeveloped in nexus research. However, both optimisation approaches benefit from dialogue with stakeholders. Recent studies illustrate how widening stakeholder interaction during the development of optimisation tools permits a sharing of values and visions that aids the exploration of possible interventions, with potential for consensus building and conflict resolution (Maier et al. 2014). Furthermore, stakeholder engagement increases the validity of evaluation in either approach by basing assumptions in grounded understandings of nexus systems (Karjalainen et al. 2013).

Finally, given the complexity of the nexus, visualisation techniques are critical to convey system state and dynamics, explore trade-offs, and communicate multidimensionality and interaction. Additionally, visualisation plays a valuable role in widening and enhancing stakeholder engagement in decision making processes, as is demonstrated in the urban design context by Isaacs et al. (2011). Various visualisation techniques have been applied in nexus research including Sankey diagrams (Bajželj et al. 2014), interactive maps (Hadka et al. 2015) and multidimensional surfaces for exploring Pareto-optimal fronts (Hurford and Harou 2014), each with their different strengths and weaknesses. Inclusion of stakeholders in the development of such tools not only benefits decision making (Hurford and Harou 2014), but also enables a better understanding of the alternative pathways for action under conditions of uncertainty that characterise nexus challenges (Hadka et al. 2015). However, it is more challenging to involve stakeholders in some modelling techniques than others and to date, there has been limited discussion regarding the most effective visualisation techniques.

There are increasing calls for decision support tools that better accommodate and illuminate the complex, nonlinear challenges that characterise the nexus (Blackhurst and Rivers 2014), and embrace the inherent interdependencies of WEF sectors (Bazilian et al. 2011). Furthermore, in recognition of the benefits of stakeholder engagement in the design process, there is growing support for their involvement in developing tools to assess and visualise nexus systems. However, studies that include stakeholders as co-creators of the knowledge contained and portrayed in such tools remains atypical. The studies discussed above demonstrate how transdisciplinary methods enhance both the quality and rigour of tools, and also increase their application in real-world contexts. Additionally, the inclusion of stakeholders offers potential to counter historical overemphasis on macro-level resource availability to provide a more nuanced representation of nexus challenges (Flammini 2014; Biggs et al. 2015; Endo et al. 2015). Furthermore, transdisciplinary approaches provide a space in which to facilitate discussion, potentially contributing to learning and the resolution of conflict between stakeholders with divergent interests (Maier et al. 2014). Thus, key to the development of tools to assess and visualise nexus challenges is the use of interactive methods that can widen participation and promote dialogue by presenting information in an engaging and functional way.

### Understanding governance and building capacity

The social and institutional dimensions of nexus systems are often overlooked in a research space dominated by large-scale technical models. Yet, understanding governance systems is essential, both to analyse nexus challenges and to design possible solutions (Hatfield-Dodds et al. 2015; Stirling 2015). Governance refers to the diffuse networks of actors (e.g., households, firms, government departments), institutions (e.g., market rules, regulations, social norms) and actions (e.g., politics, policies, behaviours) within water, energy and food systems (Pahl-Wostl 2009). Thus, research focusing on governance acknowledges the role of actors, institutions and actions in the present and future management of WEF systems. Capturing the full range of societal challenges and potential solutions around the nexus necessitates the consideration of the multiple underlying mechanisms that influence decision making in a range of spaces; and the varying motivations and visions of stakeholders in different settings. Such an appreciation benefits from engagement with actors across the WEF nexus space, throughout the research process. Here we consider briefly how the need for stakeholder engagement is articulated within at least three discourses on nexus governance, namely around institutions, framing, and agency.

First and foremost, nexus challenges are frequently understood as a consequence of institutionalisation within WEF systems, limiting the potential for ‘nexus thinking’ (Halbe et al. 2015). Within government, for example, the compartmentalisation of water, energy and food systems within departmental silos creates the potential for unintended impacts between systems (Sharmina et al. 2016), a situation exacerbated by institutionalisation of specific types of knowledge, expertise and methods within decision making processes (Kuzemko, 2014). Similarly, there is a tendency for scholars to work within, rather than across research disciplines (Brand and Karvonen 2007). Consequently, there are calls for nexus studies that employ inter-, multi- and transdisciplinary methods to examine institutional constraints within the nexus (Howarth and Monasterolo 2016).

Second, framing effects how policy intentions are operationalised in responses to nexus challenges. Numerous possible framings exist for nexus issues (e.g., security, dependency, scarcity, risk), each of which lead to different understandings of the problem, and effect preferences for solutions (Halbe et al. 2015; Stirling 2015). For example, while early framings of the nexus emphasised the securitisation of water, energy and food resources (e.g., Hoff, 2011), such narrow conceptualisations risk closing down discourses around broader objectives such as wellbeing, equity and justice (Stirling 2015). However, there remains a balance between the desire to ‘open up’ nexus analyses through stakeholder engagement, and recognising the limits caused by an unequal distribution of resources and agency among actors (Lele et al. 2013).

Finally, the agency of groups of actors is an issue of particular relevance to understanding processes of change and continuity in nexus systems. Whether conceptualised in terms of system transition or societal transformation, understanding innovation necessitates reflection on how agency is distributed and exercised among actors (Geels et al. 2015). Existing frameworks, however, struggle to accommodate the complexity of governance regimes, whilst also retaining some level of transferability beyond immediate case studies, and there are calls for approaches that embrace complexity and recognise context dependency of governance arrangements (Pahl-Wostl et al. 2010). This challenge is amplified for nexus research, as the interconnections between governance regimes in interlinking sectors become increasingly important. Only by engaging stakeholders from throughout innovation systems (i.e., not only entrepreneurs but also incumbents, knowledge brokers, policymakers, intermediaries and civil society) can one hope to represent processes of change within the nexus.

Understanding how underlying governance systems variously constrain, enable and direct the scale and speed of change is necessary to understand how innovations might be scaled up to enhance their impact. Though analytical approaches to understand the structures and processes that support or hamper innovation exist (Hekkert et al. 2011; Mitchell 2014), application has not extended far into the WEF nexus space. Furthermore, the inclusion of stakeholders in nexus research not only allows the framing of nexus challenges to be better understood, but—depending on the modes of stakeholder engagement—provides a means of establishing a broader problem frame that better accommodates a diversity of system perspectives, thus counteracting a tendency towards siloed governance systems (Pahl-Wostl 2009). Though different problem frames may be neither wholly reconcilable nor additional, pluralism extends the scope of management options and possibilities, increasing the opportunity for innovative and appropriate design of policies and interventions (Bizikova et al. 2013, Middleton et al. 2015, Hussey and Pittock, 2012, Fao, 2014). Thus, depending on the nature of stakeholder engagement, transdisciplinary research can provide a platform for social and institutional learning that supports the transformation of governance systems themselves (Pahl-Wostl et al. 2012; Halbe et al. 2015).

### Accounting for multi- and inter-scalar relationships

The notion of scale is of significant importance to nexus research. In addition to the consideration of WEF impacts at a range of scales, the scales that matter to each component systems vary in importance. For example, water supply and sewerage systems tend to be more localised than food supply systems, that have extensive global supply chains for both inputs (e.g., fertiliser) and outputs (i.e., food). However, the effects of WEF systems may be experienced on different scales to that which they are managed, for example, the embedded water in food systems (Kumar and Singh 2005) or the emissions associated with production mean that localised patterns of consumption have global impacts (Bradley et al. 2013). Furthermore, compared to these incumbent systems, innovations within the nexus tend to be more localised social or technical experiments. In addition, there are methodological issues related to scale that arise with regards to the scope and boundaries of any research (for example, the availability, completeness and granularity of data that are available at different scales). These are, however, amplified by the complexity and multiplicity of nexus research. These multi-scalar interactions are a defining characteristic of the nexus, but are under-accounted for in methodological discussions.

The WEF nexus has been studied at the wide range of scales. For example, at the smaller scale Davies and Doyle (2015) and Watson et al. (2016) studied individual households, Leung Pah Hang et al. (2016) local area, and Macknick et al. (2012) and Clemmer et al. (2013) administrative areas. However, perhaps more common is research into the national scale interactions and impacts of nexus systems, either to understand nexus issues within a single country (e.g., Conway et al. 2015; Hatfield-Dodds et al. 2015; Tidwell 2016), or to compare between nations (e.g., Mushtaq et al. 2009). Given the importance of water within the nexus, and the distinct socio-physical geography of rivers, another common scale of research is that of the river basin—some of which are located within a single country (e.g., see Lautenbach et al. 2012) and others transboundary (e.g., see Belinskij 2015)—where it is thought that nexus research can add a new dimension to integrated water management (Granit et al. 2012; Kibaroglu and Gürsoy 2015). At the largest scale, the global impacts of existing WEF systems have been examined (e.g., Khan and Hanjra 2009) and used to inform high-level strategic visions for addressing overarching challenges such as climate change or sustainable development (Flammini 2014). However, in practice, global scale research relies upon data that are often only available at a regional or national level (Ferroukhi et al. 2015), such that global level research may be considered more akin to an almagamation of smaller-scale models.

Temporal scales are similarly challenging. Studies that look out to 2050/2100, for example, are often poorly equipped to understand the short- and medium-term impacts of policy and management activities. Similarly, analysing short-term impacts can distract from management measures with long lead times that are required to address things such as climate change (both with regard to CO_2_ and non-CO_2_ greenhouse gas emissions (Bows-Larkin et al. 2014)), or risk locking in unsustainable management agendas (e.g., Welfle et al. (2014)). Consequently, a long-term outlook is an essential part of nexus thinking, but addressing nexus issues requires short- and medium-term goals (Sharmina et al. 2016; Yang et al. 2016).

It is important that research acknowledges the multi- and inter-scalar dimensions of nexus challenges, as the selection of scale in nexus research has implications for how nexus challenges and potential solutions are framed. For example, conflicts of interests and contextual differences may either be disguised or revealed through different boundary choices, which may also render potential solutions inappropriate or not (Fam et al. 2015). Swyngedouw (2010), for instance, is critical of the global change research agenda, as it prioritises large-scale urgent action at the expense of addressing local issues, such as those related to environmental and procedural justice (see also Scott et al. 2011). Similarly, focussing on smaller scales may result in the study ‘drowning’ in empirical data, whilst being unable to draw links between case studies or produce actionable understanding, yet at other times, this may be essential for understanding contextual specifics (Ang 2011).

Scale presents an additional layer of complexity when seeking to engage with stakeholders, as the priorities of stakeholders at different scales may be a source of conflict. However, the selection of methods that provide a platform for negotiation of conflict and collaboration (Pittock et al. 2015) may aid in the identification of scalar issues, trade-offs or interdependencies, and also aid in the resolution of conflicting interests. For example, Schreiner and Baleta (2015) illustrate how different interpretations of policies between different countries and at different scales present challenges for effective implementation.

Finally, it is worth noting that sometimes the purpose of nexus research is to understand potential for upscaling, as is the case for Stepping Up. In these cases, it is not uncommon to consider the implications of specific innovations, policies or case studies to understand the implications for upscaling or processes through which upscaling may occur. The inclusion of stakeholders in research enables a clearer understanding of the dynamics and disconnects in multi-scalar systems, including governance structures, by allowing a broader appreciation of challenges faced by actors at different levels.

### Exploring implications of future change

Much nexus research is intrinsically future-focussed; designed to mitigate and adapt to future resource management challenges. Consequently, future challenges—climate change, population growth or declining productivity—often form part of the research problem. Furthermore, it is necessary to account for the dynamic context of WEF systems when examining impacts. However, there are methodological challenges involved in characterising the future social, technological and environmental conditions within which nexus systems will in future. As a result, though it is common to acknowledge the challenge such changing circumstances pose to long-term management, it is as yet uncommon that research attempts to assess how the implications of future change for WEF nexus impacts and their management. For instance, Beddington (2009) highlights the ‘perfect storm’ of climate change, growing population and rising demand for energy, food and water, yet doesn’t propose methodological approaches for assessing how such changes will effect nexus systems. Here we consider existing techniques to understand future change, and the benefits and challenges of transdisciplinary research.

Perhaps the most common method for understanding the conditions of future systems are forecasting techniques, which are used to extrapolate plausible pictures of the future from current and historical trends. These are pervasive quantitative methods that characterise future conditions based on observations of the present, often focusing on a specific sector or region. However, it is increasingly well-recognised that projections under conditions of substantial uncertainty the predictive power of forecasting techniques diminishes (Dreborg 1996; Quist and Vergragt 2006). Consequently, for research concerning the WEF nexus, where the future contains multiple sources of uncertainty, forecasting risks ascribing probability and causality inaccurately. In particular, the dependence of future conditions on decisions yet to be made by a multitude of actors in numerous sectors, and the uncertain consequences of these actions means that developing models and algorithms to forecast future conditions has questionable value (Swart et al. 2004).

Scenario planning methods, in contrast, offer scope to investigate the possible implications of changes that depart from existing trends without assuming any power of prediction (Swart et al. 2004). Scenario approaches are designed to understand the implications of possible future changes—such as to society, technology, economy and climate—and can be qualitative and/or quantitative in nature. It is increasingly common to involve stakeholders in scenario development (see Kishita et al. 2016 for review), to refine assumptions and to understand the implications of alternative scenarios. Backcasting is one scenario method, which involves “working backwards from a particular desired future end-point” (Robinson 2003, p. 842) to allow stakeholders to explore futures despite fundamental uncertainty. Backcasting entails two processes: visioning—a process of defining desirable futures—and an analysis of the processes and actions via which these visions might be achieved. Backcasting has been extensively applied in the literature on climate change, for example, to identify different possibilities for future energy systems, and to articulate possible pathways towards these future energy systems (Mander et al. 2008). However, as yet the majority of backcasting studies remain focussed on a single resource sector; see Anderson et al. (2008) and Foxon (2013) on energy; Bows et al. (2012) on food; and Atkins (2013) on water.

There are as yet few scenario studies that explore the nexus of WEF systems; however, some recent studies illustrate the value of stakeholder participation in backcasting processes and scenarios research. Stakeholder involvement creates a space for collaboration that can develop capacity for nexus thinking (Davies et al. 2012), and provide orientation and guidance for planning through uncertainty (Quist et al. 2011; van Vliet and Kok 2014). Furthermore, adequate engagement with stakeholders in developing and interrogating scenarios is shown to aid acceptability of the visions’ scenarios to describe (Soste et al. 2015), and enhance the usability and relevance of the outputs (Bows et al. 2012). Thus, scenario development can provide a vehicle for transdisciplinary learning (Berkhout et al. 2002). Nevertheless, scenario analyses pertaining to the nexus remain uncommon, and there are few published examples of studies that identify and analyse the interdependencies between the water, energy and food domains within a changing context. This is unsurprising, as the inherent subjectivity and uncertainty involved in choosing which future scenarios to explore, coupled with the complex nature of the nexus, calls for new and innovative methodological approaches. Thus, there is substantial scope to involve stakeholders to understand the implications of future change in the nexus space.

### Summary

While a considerable body of literature recognises that future pressures will create new tensions and co-benefits across the nexus, it seems likely that without new transdisciplinary approaches, there will continue to be poor coordination in addressing challenges across the water food and energy domains. With institutional fragmentation being maintained, there risks a lack of opportunities for delivering stakeholder-informed and grounded decision making that can meaningfully address society’s grand challenges. The previous sections make the case for stakeholder engagement in a range of important aspects of nexus research. The methodology designed for the Stepping Up project, which is described in Sect. 3, aims to address these issues, embedding stakeholder knowledge throughout a mixed method research design. This transdisciplinary approach is designed to produce research output both nuanced enough to account for the intricacies of implementing and scaling nexus innovations, and also to develop an understanding of the potential impacts of management actions on a larger scale.

## A transdisciplinary approach for researching nexus innovations

The remainder of this paper outlines the methodological approach developed in Stepping Up. The approach follows the definition of transdisciplinarity set out by Stock and Burton (2011), incorporating stakeholder knowledge alongside various academic disciplines. Stepping Up aims to understand the processes of implementing and scaling up innovations in the nexus space, and to take account of the dynamic context in which innovations exist, including a decarbonising energy system, increasing resource demand and a changing climate. Thus, the approach described provides the latitude to address transdisciplinary research questions around how systemic change could feasibly be accelerated. It is argued that no single model exists, or could exist, that can wholly incorporate and integrate all of the elements of the nexus described previously, and this paper is not proposing such a model. It is, however, presenting a new methodological approach that aims to test this complex system by sensitively linking quantitative models with insights derived from interviews, case studies and workshops to explore different elements of the project aims. The research relies on stakeholder involvement throughout to deliver synthesised insights at the water–energy–food nexus.

**Fig. 1:**
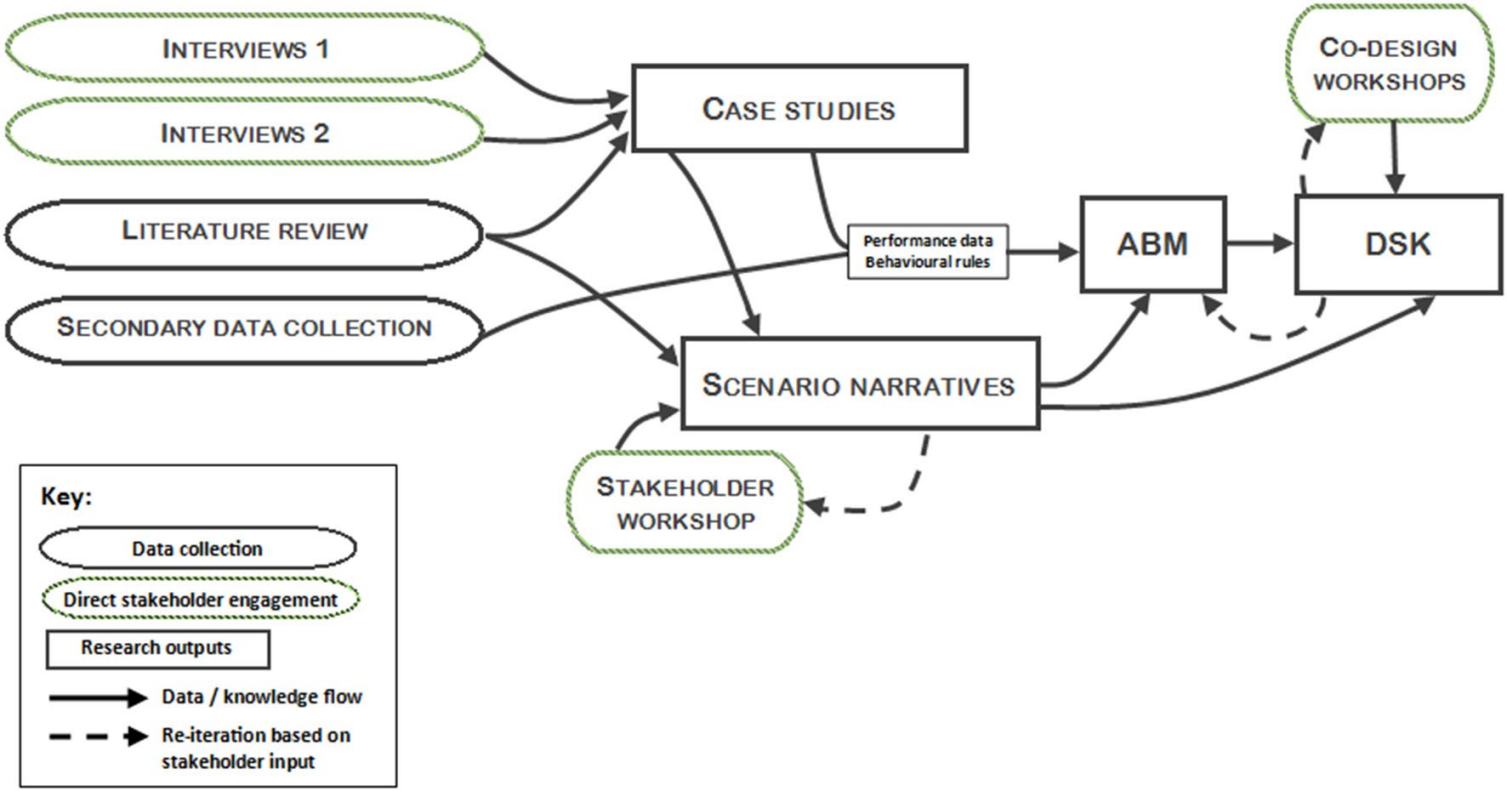
Overview of transdisciplinary research design

Figure 1 provides a simplified schematic overview of the project’s research design, illustrating the connections between different research methods employed, and in particular demonstrating how stakeholder knowledge are integrated in the research. Supported by interviews (detailed in Sect. 3.2), case studies provide rich insights on three social and technological innovations in the nexus space, as they are implemented at a range of different scales. The analysis of these case studies provides information on the context and performance of innovations, and data to inform the design of behavioural rules for an Agent Based Model (ABM) (Sect. 3.3) and scenario narratives (Sect. 3.4). Insights derived from stakeholder workshops inform various stages of the research, particularly the development of scenario narratives and a Decision Support Kit (DSK) (Sect. 3.5), which builds upon the research findings throughout the project to generate, and communicate, understanding of the impact of innovations under a range of future scenarios.

### Case studies: analysing the context and conditions of nexus innovations

In seeking to attain a breadth of understanding of potential innovation in WEF systems, the project team identified three innovations on which to focus: anaerobic digestion (AD); insects as a source of protein for humans and/or livestock; and the redistribution of surplus food. These three case studies are united as potentially beneficial means of re-appropriating the value of wastes arising throughout food supply chains, thereby avoiding landfill and waste of embodied energy and water. The selection of these innovations was informed by a review of both academic and grey literature and guided by participatory observation at various events to identify innovations with transdisciplinary interest. Each represents a socio-technical innovation in the nexus space with perceived benefits for long-term sustainability, as they offer opportunities to mitigate some of the negative environmental and social impacts of the agri-food system in its present configuration. Their varying levels of maturity and implementation offer opportunities to understand the processes of, and potential benefits resulting from, innovation in WEF nexus systems, and raise questions around systemic or step-change.

Multiple UK case studies (i.e. instances of innovation experiments) were identified for each innovation in order to reflect diversity in terms of geography, application and scale, and the different implications and challenges that such diversity brings. The three innovations vary in their levels of maturity in the UK, and enjoy varying degrees of policy support. For example while AD is relatively well established, though with considerable potential for further expansion (National Grid 2016), the potential applications of insect proteins remain relatively underexplored (van Huis et al. 2013). The case study approach thus sought to capture the dynamics of innovation processes in different contexts, to understand the conditions that support, direct and constrain processes of scaling-up, including both intrinsic (e.g., organisational capacity) and extrinsic factors (e.g., existing policy landscape) affecting case studies.

### Stakeholder interviews: unpacking governance systems

Semi-structured interviews are the principal method for case-study development, and can be broadly divided into two groups. First, interviews with innovation entrepreneurs provide a means to develop an understanding of the context specific experiences of innovation in each case study (Interviews 1 in Fig. 1). These initial interviews are sampled purposefully, identifying key actors within the innovation space to understand the processes that influence innovation in each case study. Interviews are semi-structured allowing the interviewee to guide the content towards themes they personally identify as being important (Longhurst 2009). Following the initial interview, at least one additional interview with each innovation-entrepreneur is conducted to explore cross-cutting themes. Interview data are complemented by a secondary data gathering exercise in which publically available information and that provided by interviewees (for example, on energy output of AD plants, and their location), is reviewed and collated to inform the Agent Based Model (see Sect. 3.3).

To supplement the initial purposeful sampling, snowball sampling is used, whereby additional interviewees are identified through the existing sample to extend the interview coverage (Robson 2002). This second stage of interviewing provide a means of understanding the institutional norms and experiences of actors involved in the innovation processes including, for example, regulatory bodies, local authorities and trade bodies (Interviews 2 in Fig. 1). These interviews provide further insight into the processes of innovation, enabling the complex interactions between organisations influential in the nexus to be more fully understood. The number of interviews associated with each innovation or case study vary, with interviewees added until the new themes emerging within the interview is substantially diminished, a point referred to as saturation (Guest et al. 2006). For example, many more interviews have been carried out towards the AD study compared to insect protein, where the pool of actors—and their subsequent experiences—is relatively small in comparison, and the themes emerging within the interviews are more limited.

### Agent based model

Agent based modelling (ABM) is used to understand the processes and implications of scaling innovations from their existing state to a national scale, and draws on insights derived from the case studies to ground the model assumptions in stakeholder expertise. ABM disaggregates systems into individual components (agents), and defines their behaviour rules to simulate their actions in a defined landscape. Therefore, ABM is a bottom-up approach to modelling the evolution of a specified system, where the overall behaviour of a system emerges from the behaviours and interactions of autonomous agents (Batty et al. 2012). Agent Based Models are adept at modelling complex, nonlinear systems, as they enable the definition of the system and agents with a relatively simple set of rules. Heterogeneity may also be represented, as bespoke parameters may be established to define agent subgroups, effecting the resulting overall agent population.

The method used in Stepping Up implements and extends the framework proposed by Bazilian et al. (2011) in the context of the UK with the development of an Agent Based Model. Bazilian et al. (2011) are critical of the gaps in other integrated modelling approaches, highlighting the single system and single resource focus, the lack of data and methodological components, overly simplified geographical representations, unrealistic scenarios and lack of decision support. A modelling framework for water, energy and food is proposed for Stepping Up that addresses these gaps and also the challenge of using a systems approach, recognising that technology options can affect multiple resources and that policy across systems requires harmonisation and integration to mitigate contradictions. In Stepping Up, both qualitative and quantitative data attained through the case study interviews and secondary data collection are integrated in the model, defining the behaviour rules of agents and performance of the innovations [a full method is reported in Haltas et al. (2017)]. Scenario narratives provide the basis to test the influence of varying different assumptions in the ABM for the diffusion and performance of innovations. Thus the Stepping Up’s approach to ABM grounds model assumptions in stakeholder understanding to specify agent responses to future change.

ABM provides a means to integrate some of the research findings from across Stepping Up research. The information and physical resource exchange between stakeholders across WEF systems is modelled using the ABM to understand the significance of different governance arrangements and the implications of different future scenarios (see Sect. 3.4). Further the simulation outputs will provide input into the Decision Support Kit, demonstrating the simulated effects for water, energy and food systems, and also on the inter-connections between these systems, identifying points of stress, where demand and supply are proximate or compromised.

### Scenarios: understanding future context of nexus challenges

Scenarios are used in Stepping Up to explore how innovation diffusion and impacts may differ in light of changes in climate (e.g., precipitation rates and temperature change) and social and technological changes in production-consumption systems (e.g., changing patterns of demand for energy, food and water). Though change is inherent in dynamic systems, and we therefore know that the nexus system will differ from today, the extent and nature of changes experienced is uncertain. Thus scenarios provide a basis to explore various possible futures to understand the characteristics of the world in which the selected nexus innovations might exist in the future. There are numerous methods that might be used to engage stakeholders in scenario development. The method used in Stepping Up enables coherent high-level narratives to be interrogated by innovation-entrepreneurs and other stakeholders, to ground scenario narratives in their experiences.

The scenario narratives are based in existing scenarios literature, where various studies were found relating to water, energy or food. The insights and assumptions from these studies were synthesised and, alongside data from the case study interviews, are used to develop three scenario narratives. Narratives provide qualitative depictions of the alternative conditions in which our innovations might exist, describing the overarching changes in climate and production-consumption systems (e.g., the state of the energy system, future food waste streams, changing patterns of demand, policy landscape, and characteristics of trade arrangements).

Following their initial development, scenario narratives provide the basis for a stakeholder workshop that is used to refine the assumptions being made; explore the implications of these high-level narratives for the specific case studies and innovations identified; and understand the actions, policies and interventions that might be aligned with different futures. This process is designed to enrich the scenarios, making them more robust and relevant to the real-world challenges faced by those engaged in nexus innovations, and to develop critical detail regarding the translation of high-level narratives. The scenario narratives also provide the basis for visualising the future of innovations, and conducting a participatory backcasting process to examine pathways to enable effective innovation in a changing context. Thus there are numerous learning opportunities stemming from the scenarios, both for the researchers and stakeholders involved in Stepping Up. Indeed existing evaluations of scenario analyses illustrate that the process of developing visions and negotiating pathways towards them offers more opportunities for learning than any final suite of scenarios or output developed (Quist and Vergragt 2006). Participatory scenario development in particular facilitates the interaction between stakeholders from different backgrounds and with researchers, that is not only an opportunity to change understandings and framings of the problem (Davies et al. 2012), but to agree potential solutions (Cornell et al. 2013), and enhance capacity for action (van der Heijden et al. 2002).

In addition, the scenarios provide logic and data to inform and vary parameters in the ABM, allowing the effects of changes to be visualised (see Sect. 3.5). For example, logics within the scenario narrative can be used to identify existing data on climate change (e.g., from UKCP09) to input data to hydrological and land-use components of the ABM at case study sites. Data on other system dynamics, for example changes in food waste streams, energy systems (e.g., extent of decarbonisation or decentralisation), and water demand can be found in existing research and interpreted using the scenario narratives to inform the ABM so that the contingencies of innovation diffusion and impact can be better understood.

### Decision support kit

The decision support kit (DSK) integrates and makes use of interdisciplinary, cross-sectoral data and presents the results in a way that is easily interpreted by stakeholders. The DSK will be developed with the intended users at the centre and draw in tacit knowledge from a wider stakeholder base. The users of the DSK are those who can influence change towards a lower impact WEF system via the identified innovations e.g., food and drink industries and local and regional government. The many ways in which potential innovations may be scaled up and/or replicated is reflected in the diversity of potential end users. This raises design challenges and will be addressed through a co-design workshop. This approach, facilitated by the transdisciplinary focus of the research, will ameliorate some of the criticisms raised by Rose et al. (2016); that the majority of existing DSK have limited reach and are not useful.

The envisioned principal components of the DSK are: (1) WEF indicators which are used to describe both a baseline WEF state and also a predicted WEF state reached via simulation. (2) A tool for Multi Criteria Decision Analysis (MCDA) used to identify the ‘best fit’ niche innovation if more than one alternative exists. (3) Data visualisation that depicts changes in WEF state together with uncertainty.

From a defined set of WEF indicators, a context specific subset will be drawn. These indicators will afford a baseline assessment of the context-dependent WEF state. Using information gathered through the case studies, and relevant literature, the adoption of innovations will be simulated, using ABM, to evaluate the impact on the WEF state. Where multiple innovations are applicable, MCDA tools provide a means to identify and compare value. The MCDA method will be selected based on the needs and requirements of the users at the co-design workshop. Methods of data visualisation will be used to facilitate understanding of the invoked system changes. Running the Agent Based Model many times with perturbations will provide a measure of uncertainty which will be presented to the decision makers.

## Conclusion

This paper has explored the way in which stakeholders have been embedded in nexus research to date, and has argued that a transdisciplinary approach can improve the quality of research in various ways. Instrumental value through the inclusion of stakeholder knowledge, expertise and data, opportunities for learning and capacity building through discussion between stakeholders and improvement in terms of the usability, uptake and impact of research outputs through the co-development of visualisation approaches and decision support tools are all important drivers for significant upstream stakeholder engagement. It is argued that the process of stakeholder engagement where different experiences, framings and priorities are discussed and debated offers significant value in and of itself, rather than being a route only to improved outputs. In addition, stakeholder engagement in the nexus is presented as being central to understanding social and institutional dimensions of the nexus, particularly in terms of dynamism and inertia that characterise governance frameworks.

In the Stepping Up project, stakeholder engagement plays a crucial role in the method from the outset. Case studies of nexus innovations are taken as the starting point (anaerobic digestion, insects as a source of protein for humans and/or livestock and the redistribution of surplus food), with interviews providing both innovation performance data and behavioural rules for the Agent Based Model. The Agent Based Model integrates findings across the project and connects case study innovations to wider regional and national nexus scales, facilitating examination of the potential for scaling-up of these innovations. The implications of future change in terms of hydrology, land-use and food, climate impacts, governance, energy policy, agricultural policy and infrastructure are included in the analysis through exploratory scenarios with stakeholder interviews and a workshop shaping the selection of these. The appropriate indicators and visualisation approaches for stakeholders are developed through co-design and testing workshops. It is argued that this approach allows for the development of a detailed and practical understanding of the multiple contexts, enablers and barriers within which changes to improve the sustainability of the nexus could happen and the potential for scaling-up these specific innovations in the future.

This proposed new methodology is ambitious and presents various technical and integrative challenges, however, in line with our view on the value of the process of stakeholder engagement, the process of developing and delivering this approach seems likely to yield valuable transdisciplinary learning in nexus.
